# Lifetime Exposure to Depression and Neuroimaging Measures of Brain Structure and Function

**DOI:** 10.1001/jamanetworkopen.2023.56787

**Published:** 2024-02-19

**Authors:** Xinyi Wang, Felix Hoffstaedter, Jan Kasper, Simon B. Eickhoff, Kaustubh R. Patil, Juergen Dukart

**Affiliations:** 1 School of Biological Sciences and Medical Engineering, Child Development, and Learning Science, Key Laboratory of Ministry of Education, Southeast University, Nanjing, China; 2 Institute of Systems Neuroscience, Medical Faculty, Heinrich Heine University Düsseldorf, Düsseldorf, Germany; 3 Institute of Neuroscience and Medicine, INM-7: Brain and Behaviour, Research Centre Jülich, Jülich, Germany

## Abstract

**Importance:**

Despite decades of neuroimaging studies reporting brain structural and functional alterations in depression, discrepancies in findings across studies and limited convergence across meta-analyses have raised questions about the consistency and robustness of the observed brain phenotypes.

**Objective:**

To investigate the associations between 6 operational criteria of lifetime exposure to depression and functional and structural neuroimaging measures.

**Design, Setting, and Participants:**

This cross-sectional study analyzed data from a UK Biobank cohort of individuals aged 45 to 80 years who were enrolled between January 1, 2014, and December 31, 2018. Participants included individuals with a lifetime exposure to depression and matched healthy controls without indications of psychosis, mental illness, behavior disorder, and disease of the nervous system. Six operational criteria of lifetime exposure to depression were evaluated: help seeking for depression; self-reported depression; antidepressant use; depression definition by Smith et al; hospital *International Statistical Classification of Diseases and Related Health Problems, Tenth Revision* (*ICD-10*) diagnosis codes F32 and F33; and Composite International Diagnostic Interview Short Form score. Six increasingly restrictive depression definitions and groups were defined based on the 6 depression criteria, ranging from meeting only 1 criterion to meeting all 6 criteria. Data were analyzed between January and October 2022.

**Main Outcomes and Measures:**

Functional measures were calculated using voxel-wise fractional amplitude of low-frequency fluctuation (fALFF), global correlation (GCOR), and local correlation (LCOR). Structural measures were calculated using gray matter volume (GMV).

**Results:**

The study included 20 484 individuals with lifetime depression (12 645 females [61.7%]; mean [SD] age, 63.91 [7.60] years) and 25 462 healthy controls (14 078 males [55.3%]; mean [SD] age, 65.05 [7.8] years). Across all depression criteria, individuals with lifetime depression displayed regionally consistent decreases in fALFF, LCOR, and GCOR (Cohen *d* range, −0.53 [95% CI, −0.88 to −0.15] to −0.04 [95% CI, −0.07 to −0.01]) but not in GMV (Cohen *d* range, −0.47 [95 % CI, −0.75 to −0.12] to 0.26 [95% CI, 0.15-0.37]). Hospital *ICD-10* diagnosis codes F32 and F33 (median [IQR] difference in effect sizes, −0.14 [−0.17 to −0.11]) and antidepressant use (median [IQR] difference in effect sizes, −0.12 [−0.16 to −0.10]) were criteria associated with the most pronounced alterations.

**Conclusions and Relevance:**

Results of this cross-sectional study indicate that lifetime exposure to depression was associated with robust functional changes, with a more restrictive depression definition revealing more pronounced alterations. Different inclusion criteria for depression may be associated with the substantial variation in imaging findings reported in the literature.

## Key Points

**Question:**

How are the 6 operational criteria of lifetime exposure to depression associated with functional and structural neuroimaging measures?

**Findings:**

In this cross-sectional study of 20 484 individuals with a lifetime exposure to depression and 25 462 healthy controls in the UK Biobank, robust functional alterations but not structural alterations were observed with more restrictive criteria of depression.

**Meaning:**

Findings of this study suggest that different inclusion criteria for depression may be associated with the substantial variation in imaging findings described in the literature.

## Teaser

This cross-sectional study evaluates the association between different definitions of lifetime depression and brain functional and structural alterations in individuals with major depressive disorder.

## Introduction

Depression is a common health condition characterized by low mood, loss of interest, and feelings of excessive guilt.^1^ The lifetime prevalence of depression is approximately 11%, with a higher prevalence in women.^2^ Exposure to major depressive disorder (MDD) is associated with reduced quality of life and increased risk of suicide and self-wounding.^3,4^ Understanding the neurobiological mechanisms underlying depression is a crucial aspect of developing improved therapeutic options and minimizing adverse outcomes.

Numerous functional and structural magnetic resonance imaging (MRI) studies have tested for neurobiological mechanisms of MDD, reporting a variety of MDD-related alterations, such as hippocampal volume reductions and increased amygdala activity during emotional tasks.^5^ In contrast, neuroimaging meta-analyses and large-scale projects revealed less convergent findings or small effect sizes for most of the evaluated neuroimaging modalities.^6,7,8,9^ When present, the identified brain alterations did not allow for reliable differentiation between patients with MDD and healthy controls, with accuracies being only marginally above chance level.^10^ The small effect sizes and lack of differentiation point to currently limited diagnostic value of respective MRI modalities.^10,11^ A confounding factor in this regard, which has been largely ignored to date, is the difference in definition of depression applied across various cross-sectional and longitudinal studies. Despite this limitation, these studies point to the existence of some MDD-related brain alterations that are either predisposing factors or a consequence of MDD diagnosis or treatment. However, it remains largely unknown whether and how far these alterations persist and whether prior exposure to depression affects brain function and structure later in life.

We used data from the UK Biobank^12,13^ to systematically quantify the magnitude of structural and functional alterations associated with lifetime exposure to depression. Making use of the available in-depth phenotyping, we aimed to investigate the associations between 6 operational criteria of lifetime exposure to depression, ranging from self-reported to clinically defined depression, and functional and structural neuroimaging measures. We then evaluated the magnitude of case-control differences as manifested in brain structure and function for the different constellations of these criteria.

## Methods

Data were obtained from the UK Biobank for individuals aged 45 to 80 years and who were enrolled from January 1, 2014, to December 31, 2018. The North-West Multicenter Research Ethics Committee approved the UK Biobank cohort study, which was conducted in accordance with the Declaration of Helsinki.^14^ All participants provided written informed consent. The Heinrich Heine University Institutional Review Board approved this cross-sectional study, and the initial informed consent from the UK Biobank participants applies to this study. We followed the Strengthening the Reporting of Observational Studies in Epidemiology (STROBE) reporting guideline.

### Participants

Six operational criteria of lifetime exposure to depression are commonly used in UK Biobank depression studies: help seeking for depression; self-reported depression; antidepressant use; depression definition by Smith et al^15^; hospital *International Statistical Classification of Diseases and Related Health Problems, Tenth Revision* (*ICD-10*) diagnosis codes F32 and F33; and Composite International Diagnostic Interview Short Form score.^16^ The patients included in the study met at least 1 of these 6 criteria. Detailed descriptions of the 6 operational criteria and exclusion criteria are provided in the eMethods in Supplement 1.

To determine how exposure to lifetime depression differed in brain structural and resting-state functional measures across cumulative criteria, we first stratified all patients into 6 groups according to the number of criteria met. These 6 graded groups were classified from meeting only 1 criterion to meeting all 6 criteria. Therefore, meeting *k* criteria indicated that participants met exactly rather than at least a specific number of criteria, ensuring that the 6 groups included no overlapping participants.

Healthy controls were defined as individuals without indications of psychosis, mental illness, behavior disorder, and disease of the nervous system. The exclusion criteria for healthy controls are detailed in the eMethods in Supplement 1. Healthy control groups were defined using 2 strategies. In strategy 1, we identified a single healthy control group that included all individuals who did not meet any of the exclusion criteria. Strategy 1 ensured that all patient subgroups were compared with the same, more representative healthy control group. To control for potential differences in demographics between the healthy control population and respective depression subgroups, we treated the demographic variables as covariates.

In strategy 2, we matched the 6 healthy control groups to the 6 depression groups by age, sex, and years of education using an automated 1:1 matching process whenever possible. Strategy 2 was a traditional control group approach to better control for potential demographic confounders. We considered the results from strategy 1 (all available healthy individuals) as the primary outcome and the results from strategy 2 as the control analysis.

### Imaging Data Acquisition and Preprocessing

Resting-state functional MRI and T1-weight structural images were acquired using a 3T scanner (MAGNETOM Skyra; Siemens Healthcare) with a standard 32-channel head coil, according to available protocol. Structural images were preprocessed using SPM12, version r7770 (Functional Imaging Laboratory) and CAT12, version r1720 (Christian Gaser and Robert Dahnke),^17^ with the default settings compiled under MATLAB 2019b, massively parallelized on the JURECA high-performance computing system (Jülich Supercomputing Centre). Functional images were preprocessed using SPM12, FSL5.0,^18^ and the CONN toolbox.^19^ The scans parameters and preprocessing are provided in the eMethods in Supplement 1.

Relative gray matter volume (GMV) was computed by dividing the total GMV by the total intracranial volume. The gray matter images were smoothed using an 8-mm full-width at half-maximum Gaussian kernel. We then computed functional measures using the default settings in the CONN toolbox, including the fractional amplitude of low-frequency fluctuation (fALFF), global correlation (GCOR), and local correlation (LCOR). The fALFF reflects the local amplitude of low-frequency fluctuation (0.008 Hz to 0.09 Hz) vs the overall frequency spectrum.^20^ The LCOR is the local coherence between a voxel and its neighboring voxels.^21^ The GCOR is the mean correlation coefficient between BOLD (blood oxygen level–dependent) signals of a voxel and all other voxels in the brain.

### Statistical Analysis

We performed a 2-stage analysis to determine brain alterations using increasingly restrictive levels of definitions, ranging from meeting only 1 criterion to meeting all 6 criteria. Two-sample, 2-tailed *t* tests were used to compare each lifetime depression group with healthy controls at *P* < .05 (family-wise error–corrected for multiple comparisons at the cluster level), followed by computation of effect sizes between all constellations of lifetime depression and the single healthy control group. Data were analyzed between January and October 2022.

In stage 1 analysis, we identified clusters of voxels that differed substantially between healthy control and each depression group, from those meeting only 1 criterion to those meeting all 6 criteria. Voxel-wise 2-sample *t* tests (assuming unequal variance) were conducted using SPM12 implemented in MATLAB (The MathWorks Inc). First, a 2-sample *t* test was used to investigate group differences in voxel-wise fALFF, GCOR, LCOR, and GMV, adjusting for age, age^2^, sex, and total intracranial volume. The 6 depression groups were compared separately with 2 types of healthy control groups (strategy 1 and strategy 2). The resulting statistical maps were at a threshold of *P* < .001 uncorrected at the voxel level combined with a whole-brain voxel-wise family-wise error correction at the cluster level at a *P* < .05 threshold. Clusters that survived this correction were considered to be statistically significant. This procedure yielded 2 directional (increases and decreases) tests per imaging modality times 6 depression strata times 4 modalities, resulting in 48 tests per strategy overall.

In stage 2 analysis, to minimize the role of group sizes in the observed differences, we quantified the observed alterations using effect size measures (Cohen *d*). Cohen *d* was used to compute the mean difference between depression and healthy control groups divided by the pooled SD. For this calculation, we defined binary voxel-wise maps of significant cluster findings from the *t*-contrasts described (separate for increases and decreases). These masks, which represented regions with significant differences for the respective contrast, were referred to as mask 1 (meeting only 1 criterion) to mask 6 (meeting all 6 criteria). Theoretically, there can be 12 masks (6 groups ×2 directional *t*-contrasts) for each structural or functional measure. For each participant, we calculated the mean values in each mask for the respective structural or functional measures. We then calculated the Cohen *d* value for each mask between the 6 graded depression groups and the single healthy control group (strategy 1).

To further explore how the different lifetime depression groups and their combinations were associated with the observed differences, we computed the effect sizes (Cohen *d*) of group differences for each available combination of depression criteria and each mask. To ensure a more robust estimate of the effect size, we excluded criteria constellations with fewer than 10 available participants. To identify the criteria associated with increases or decreases of the observed effect sizes, we quantified the association of each criterion by computing the difference in effect size. Specifically, we categorized all criteria constellations into 2 groups: with the specific criterion and without the criterion. The difference in effect size was defined as the mean effect size of combinations with the specific criterion minus the mean effect sizes of combinations without the criterion. For each identified mask per modality, this resulted in 6 differences in effect size values representing the associations of the 6 criteria.

## Results

### Sample

Among the individuals with imaging data in the UK Biobank, 20 484 met at least 1 of the criteria of lifetime exposure to depression and 25 462 met the criteria for healthy controls. Of the participants with lifetime exposure to depression, 12 645 (61.7%) self-reported as female and 7839 (38.3%) as male, with a mean (SD) age of 63.91 (7.60) years and mean (SD) years of education of 16.65 (3.82). Demographic characteristics of participants for 6 operational criteria and 6 graded depression groups are shown in the Table. Group sizes differed slightly for functional and structural analyses due to different dropouts for quality control reasons (eTable 1 in Supplement 1).

**Table.** Demographic Characteristics of Participants With Lifetime Exposure to Depression

Characteristic	Total No.	Female sex, No. (%)	Male sex, No. (%)	Age, mean (SD), y	Years of education, mean (SD)
Criteria of lifetime exposure to depression					
Help seeking for depression	19 182	11 828 (61.7)	7354 (38.3)	63.91 (7.60)	16.65 (3.82)
Self-reported depression	4691	2879 (61.4)	1812 (38.6)	63.98 (7.62)	16.64 (3.82)
Antidepressant use	4222	2602 (61.6)	1620 (38.4)	63.98 (7.64)	16.65 (3.81)
Depression definition by Smith et al^15^	3166	1963 (62.0)	1203 (38.0)	63.99 (7.67)	16.65 (3.83)
Hospital *ICD-10* diagnosis codes F32 and F33	1605	990 (61.7)	615 (38.3)	64.04 (7.67)	16.67 (3.83)
CIDI-SF score	3571	2200 (61.6)	1371 (38.4)	63.99 (7.66)	16.66 (3.82)
No. of criteria met					
1	10 977	6487 (59.1)	4490 (40.9)	64.54 (7.57)	16.52 (3.90)
2	5233	3307 (63.2)	1926 (36.8)	63.62 (7.53)	16.77 (3.76)
3	2574	1691 (65.7)	883 (34.3)	63.02 (7.58)	16.77 (3.74)
4	1275	859 (67.4)	416 (32.6)	62.40 (7.55)	16.80 (3.76)
5	378	270 (71.4)	108 (28.6)	61.79 (7.70)	17.39 (3.27)
6	47	31 (66.0)	16 (34.0)	59.22 (6.76)	17.81 (2.35)

Abbreviations: CIDI-SF, Composite International Diagnostic Interview Short Form; *ICD-10*, *International Statistical Classification of Diseases and Related Health Problems, Tenth Revision*.

The healthy controls comprised 11 384 participants who self-reported as female (44.7%) and 14 078 as male (55.3%), with a mean (SD) age of 65.05 (7.80) years and mean (SD) years of education of 16.69 (3.77). The demographics of all healthy controls (strategy 1) are provided in eTable 2 in Supplement 1, and eTable 3 in Supplement 1 shows the characteristics for matched healthy controls (strategy 2). There were 63 unique constellations of the 6 criteria, ranging from meeting at least 1 criterion to meeting all 6 criteria. For example, participants meeting all 6 criteria would also be represented in any other constellation. The number of participants for 63 constellations are shown in eFigure 1 in Supplement 1.

### Structural and Functional Alterations

We found significant alterations in all functional and structural measures for most lifetime depression groups, except in patients who met all 6 criteria or met only 1 criterion when using GMV and GCOR (eFigures 2 and 3 in Supplement 1) compared with the single healthy control group. Group comparisons in functional measures revealed consistently decreased fALFF, GCOR, and LCOR in the lifetime depression groups but not GMV compared with healthy controls. Clusters of significant functional alterations covered multiple regions encompassing the prefrontal cortex, parietal cortex, middle temporal cortex, fusiform gyrus, occipital cortex, and cerebellum (*t*_15095_ = 9.59; *P* < .001) (eTables 4-6 in Supplement 1). Specifically, fALFF and LCOR alterations displayed similar spatial patterns of decreases in the bilateral precentral and postcentral gyrus. Decreased GCOR was primarily observed in the middle inferior temporal cortex, superior temporal cortex, precuneus, insula, and lingual cortex (*t*_15095_ = 7.68; *P* < .001). We found a bidirectional pattern of GMV alterations with increases in the right superior medial frontal cortex and precentral gyrus and decreases in the right hippocampus and superior temporal cortex (*t*_22389_ = 6.86; *P* < .001) (eTable 7 in Supplement 1). The outcomes of comparisons of all depression groups to matched healthy controls were largely consistent with the primary analysis (eTables 8-11 in Supplement 1).

### Differences Between Depression Groups and Healthy Controls by Observed Alterations

**Figure 1.**
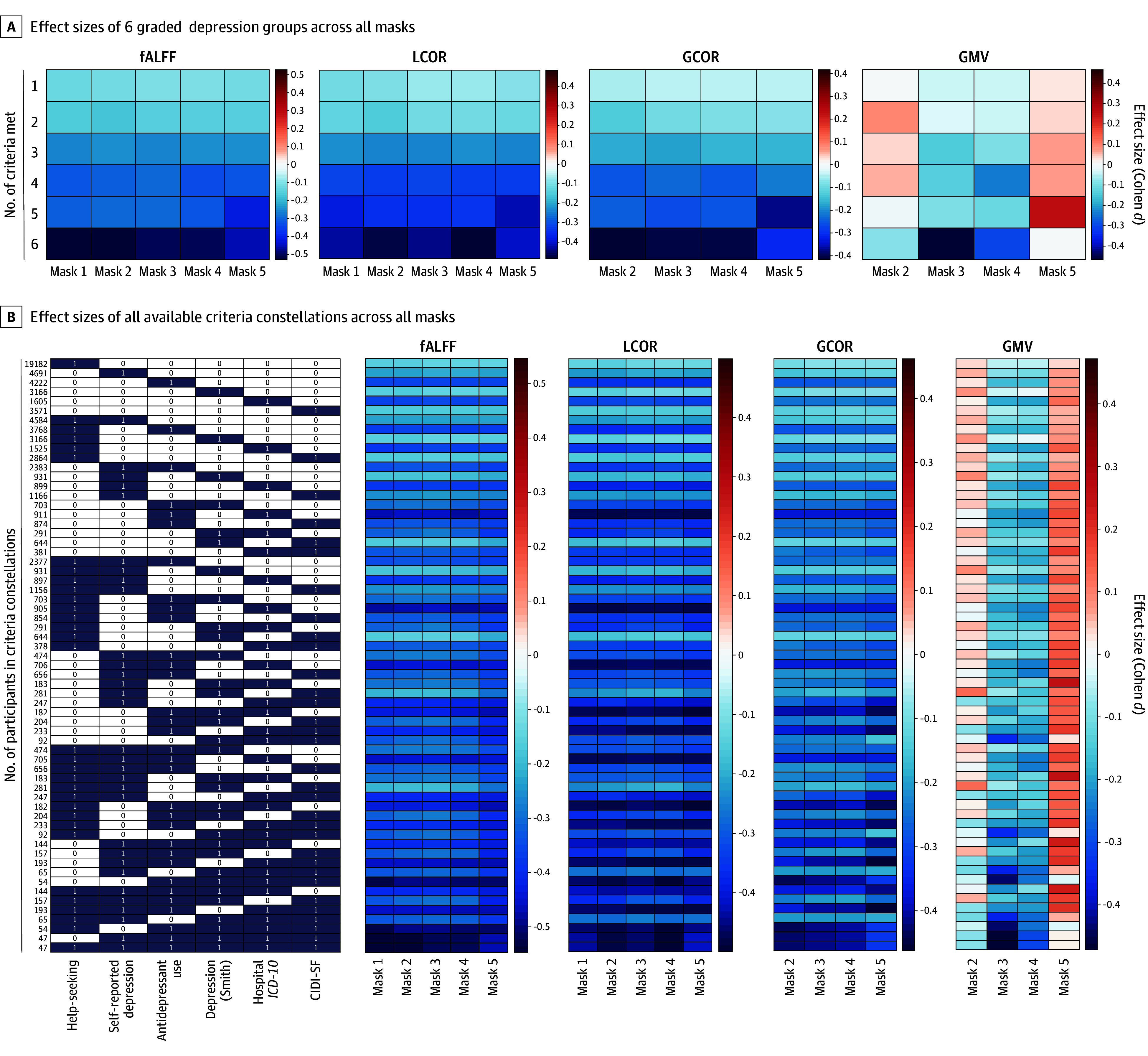
Effect Sizes for Different Lifetime Depression on the Observed Imaging Alterations A, Masks indicate the number of criteria met (eg, mask 1 indicates meeting 1 criterion; mask 5, meeting 5 criteria). Effect sizes were calculated in all depression stratum–mask pairs. B, The dark element indicates that the column contains corresponding criteria. Other blocks show the effect size values (Cohen *d*). Red indicates increased imaging measures in depression group, and blue indicates reduced imaging measures in depression group. CIDI-SF indicates Composite International Diagnostic Interview Short Form; fALFF, fractional amplitude of low-frequency fluctuations; GCOR, global correlation; GMV, gray matter volume; *ICD-10, International Statistical Classification of Diseases and Related Health Problems, Tenth Revision;* LCOR, local correlation.

Given that some of the observed imaging differences across the increasingly restrictive depression definitions may simply reflect differences in sample size, effect sizes for each criteria constellation and each significance mask derived from the group comparisons are presented in Figure 1. Effect sizes were consistently reduced in depression group for all functional measures (Cohen *d* range, −0.53 [95% CI, −0.88 to −0.15] to −0.04 [95% CI, −0.07 to −0.01]) but not for GMV (Cohen *d* range, −0.47 [95% CI, −0.75 to −0.12] to 0.26 [95% CI, 0.15-0.37]). Additionally, effect sizes increased with a higher number of criteria met for functional measures. The group that met all 6 criteria consistently showed the largest effect sizes in fALFF, LCOR, and GCOR. Effect sizes were generally lower for GMV, and only mask 3 and mask 4 from the decreased GMV displayed consistently reduced depression but overall negligible effect sizes. Results for mask 2 and mask 5 derived from the increased GMV clusters were not consistent.

### Association of 6 Criteria With Observed Alterations

**Figure 2.**
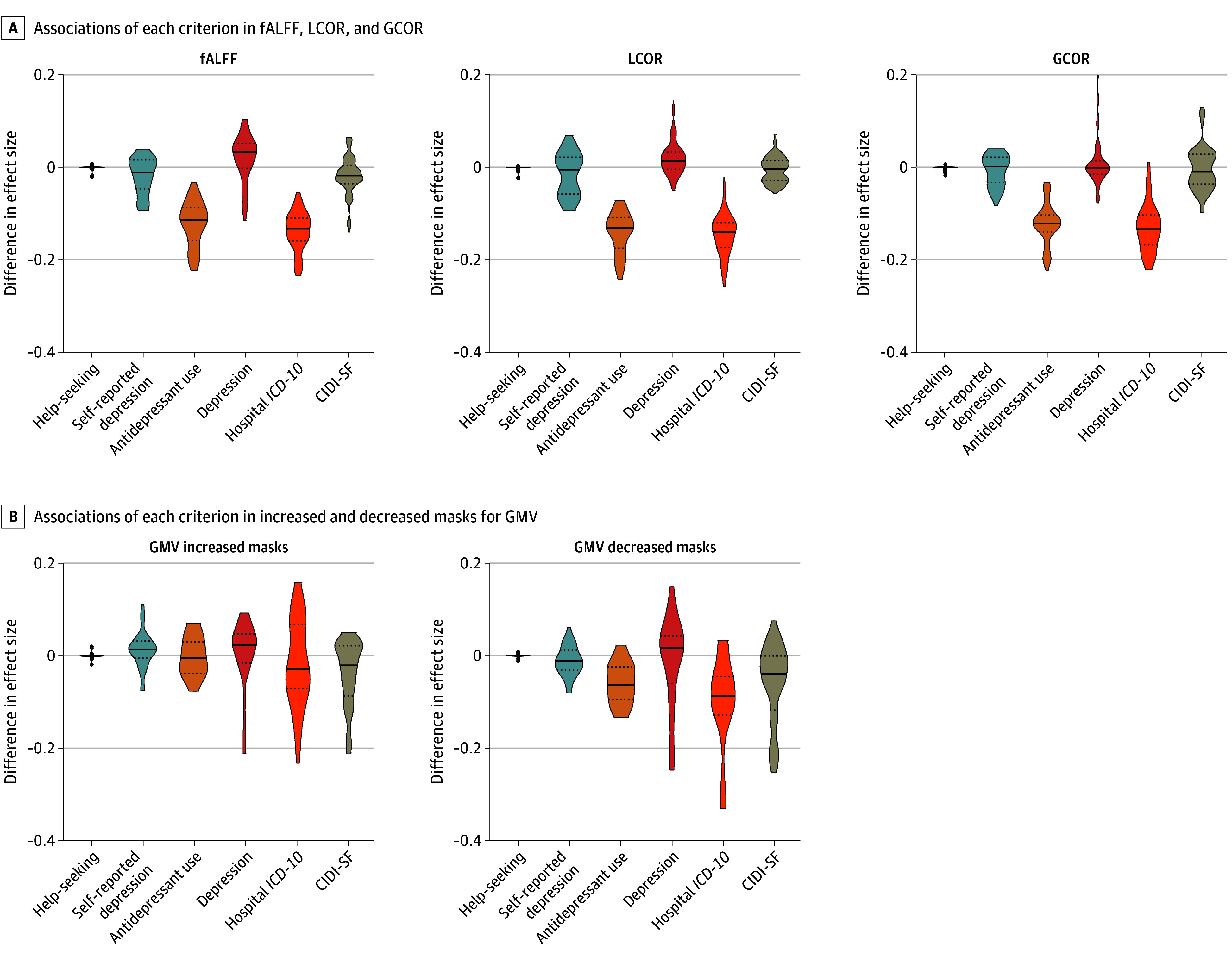
Violin Plots of Associations of 6 Criteria With Observed Alterations The extent of the violin plot is the range of difference in effect size. Solid lines indicate the median, and dotted lines indicate the quartiles of difference in effect size. Each side of the violin plot represents the kernel density estimation of the distribution shape of the difference in effect size. CIDI-SF indicates Composite International Diagnostic Interview Short Form; fALFF, fractional amplitude of low-frequency fluctuations; GCOR, global correlation; GMV, gray matter volume; *ICD-10, International Statistical Classification of Diseases and Related Health Problems, Tenth Revision;* LCOR, local correlation.

A higher absolute value in the difference in effect sizes indicates a higher association with the respective criterion. Constellations involving hospital *ICD-10* diagnosis codes F32 and F33 followed by antidepressant use displayed higher associations with the observed effect sizes for all functional measures (median [IQR] difference in effect sizes: hospital *ICD-10* diagnosis codes, −0.14 [−0.17 to −0.11]); antidepressant use, −0.12 [−0.16 to −0.10]) and for the GMV-decreased masks (median [IQR] difference in effect sizes: hospital *ICD-10* diagnosis codes, −0.09 [−0.12 to −0.05]; antidepressant use, −0.06 [−0.09 to −0.03]) (Figure 2). Higher (negative and positive) difference in effect sizes indicated associations with the respective criterion. None of the criteria displayed a consistent association for the GMV-increased mask.

## Discussion

In this cross-sectional study, we systematically tested for associations between lifetime exposure to depression and brain structural and functional measures. We found that both functional and structural alterations varied according to the different definitions of depression available in the UK Biobank, with more restrictive definitions associated with functional but not necessarily structural alterations. Specifically, decreases in local functional activity were consistent across all criteria, with hospital *ICD-10* diagnosis codes F32 and F33 and antidepressant use being associated with the observed alterations.

Increases and decreases in brain functional measures have been associated with depression in previous studies.^22,23,24,25^ In contrast to some of these findings, we observed only decreases in local activity and in local and global connectivity measures across all constellations of lifetime depression. These findings are in line with recent larger studies and meta-analyses reporting reduced functional connectivity in the default mode networks and convergent regions only for decreased functional findings in MDD.^7,26^ Despite the matching direction of change, we found only limited convergence in sensorimotor regions in terms of location of the observed alteration patterns. While differences in the samples and applied methods likely accounted for some of the discrepancies, a main difference is that this study characterized lifetime depression criteria as opposed to the more acute effects of depression as reported in the literature. The findings from this study suggest the existence of a persistent depression-related brain functional phenotype with small to moderate effect sizes.

We found that the hospital *ICD-10* diagnosis codes F32 and F33 and antidepressant use criteria were associated with the observed functional alterations in depression groups compared with healthy controls. Only limited association was observed for all other applied criteria. This observation may indicate some conceptual advantages of these 2 criteria over the other definitions of depression in the UK Biobank. We did not find similarly high effect sizes for participants meeting the Composite International Diagnostic Interview Short Form score criterion, although the screening was designed to provide lifetime information about depression and additional factors that may be associated with the observed imaging alterations. As the antidepressant use criterion is typically directly associated with a depression diagnosis, any causal interpretation of the observed alterations or conclusion about the cause or consequence of requiring medication should be limited. Despite this limitation, given that numerous treatment interventions for depression are not medication-based but psychological (eg, cognitive behavioral therapy and stimulation therapies) and that patients often refuse antidepressant treatment, these 2 criteria are not necessarily identical. The separate roles of hospital *ICD-10* diagnosis codes F32 and F33 and antidepressant use observed in the study may point to such a differential association of both variables with the observed decreases in brain functional measures. Additionally, causal interpretation is precluded in this case because the observed associations can still be attributed to either the necessity of prescribing antidepressants or the consequence of exposure to antidepressants.

We found that decreased local functional activity and synchronicity in precentral and postcentral gyrus and decreased global functional connectivity in parts of the limbic system were consistently associated with lifetime depression. The reduced local activity in sensorimotor regions may be attributed to the consequence of exposure to treatments and depression-associated vulnerability. For treatment effects, a meta-analysis found that stimulation therapy for depression altered the activity in the right precentral gyrus, right posterior cingulate, left inferior frontal gyrus, and left middle frontal gyrus.^27^ After electroconvulsive therapy, fALFF was reported to decrease in the right precentral gyrus.^28^ Additionally, antidepressant use was associated with reduced hyperconnectivity within the limbic system.^29^ Contrary to the functional measures, the findings for GMV were only partially consistent with previous studies. The observed reduced hippocampal volume is commonly reported in meta-analyses and case-control studies.^30^ The effect size of GVM alterations was substantially smaller compared with functional measures.

Overall, the findings of the present study provide evidence of the implications of different depression definitions for the observed imaging outcomes. The UK Biobank contains extensive data items associated with depression. However, the availability of multiple sources of information presents challenges in achieving consistent definitions of depression across different studies. Stricter definitions often conflict with the available sample size, but researchers might be tempted to apply fewer restrictions to putatively increase statistical power. This study showed that such an approach may become futile in neuroimaging because less restrictive definitions may lead to dilution of potential imaging alterations. Recent studies have explored the association of different depression phenotypes in the UK Biobank with genetic and cortical thickness measures.^31^ Although 1 of these studies suggested that a broader definition of depression may provide more tractable phenotypes,^32^ others recommended more restrictive definitions, suggesting that minimal phenotyping may play a role in biased potential findings by introducing conceptual differences in the selected cohorts.^33,34^ Expanding on these earlier findings, we believe that while restrictiveness is generally rather beneficial, the specific criteria should be carefully weighted and evaluated. Only 2 of the 6 applied depression criteria were consistently associated with the magnitude of the functional imaging alterations observed in the present study. These findings suggest that mere restrictiveness may become counterproductive and that the implications of each criterion need to be carefully evaluated in future research.

### Limitations

Several limitations apply with respect to the interpretation of the findings. To our knowledge, the UK Biobank is the largest available cohort to date and is biased in terms of cultural and educational background, primarily representing participants of White ethnicity.^35^ This bias may limit the generalizability of the results to other populations. Furthermore, the observed associations do not allow a causal interpretation of the findings regarding whether the observed imaging alterations are the cause or consequence of exposure to depression.

## Conclusions

This study found an association of lifetime depression with functional and structural imaging alterations. More restrictive depression definition revealed more pronounced changes. Different inclusion criteria for depression may be associated with the substantial variation in imaging findings in the literature. Each criterion warrants further study.

## Notes

Supplement 1.
eMethods.
**eFigure 1****.** The Number of Participants in Criteria Constellations **eFigure 2****.** The Cerebral Parts of the Clusters Showing Significant Functional or Structural Differences Between Different Depression Stratums and HC **eFigure 3.** The Cerebellum Parts of the Clusters Showing Significant Functional or Structural Differences Between Different Depression Stratums and HC **eTable 1.** Demographic of Six Depression Groups Involving Analyses **eTable 2.** Demographic of the Single Healthy Groups (Strategy 1) **eTable 3.** Demographic of Six Matched Healthy Groups (Strategy 2) **eTable 4.** Regions Showing Significant Differences in fALFF Between Individuals With Lifetime Depression and the Single HC Group (Strategy 1) **eTable 5.** Regions Showing Significant Differences in LCOR Between Individuals With Lifetime Depression and the Single HC Group (Strategy 1) **eTable 6.** Regions Showing Significant Differences in GCOR Between Individuals With Lifetime Depression and the Single HC Group (Strategy 1) **eTable 7.** Regions Showing Significant Differences in GMV Between Individuals With Lifetime Depression and the Single HC Group (Strategy 1) **eTable 8.** Regions Showing Significant Differences in fALFF Between Individuals With Lifetime Depression and Matched HC Group (Strategy 2) **eTable 9.** Regions Showing Significant Differences in LCOR Between Individuals With Lifetime Depression and Matched HC (Strategy 2) **eTable 10.** Regions Showing Significant Differences in GCOR Between Individuals With Lifetime Depression and Matched HC (Strategy 2) **eTable 11.** Regions Showing Significant Differences in GMV Between Individuals With Lifetime Depression and Matched HC (Strategy 2)
eReferences

Supplement 2.
Data Sharing Statement
