# Supplementary material for: Lifetime Exposure to Depression and Neuroimaging Measures of Brain Structure and Function
Source: JAMA Netw Open. 2024 Feb 19;7(2):e2356787. doi: 10.1001/jamanetworkopen.2023.56787 (PMC10877455; doi:10.1001/jamanetworkopen.2023.56787)
Supplement: Supplement 1. — eMethods. eFigure 1. The Number of Participants in Criteria Constellations eFigure 2. The Cerebral Parts of the Clusters Showing Significant Functional or Structural Differences Between Different Depression Stratums and HC eFigure 3. The Cerebellum Parts of the Clusters Showing Significant Functional or Structural Differences Between Different Depression Stratums and HC eTable 1. Demographic of Six Depression Groups Involving Analyses eTable 2. Demographic of the Single Healthy Groups (Strategy 1) eTable 3. Demographic of Six Matched Healthy Groups (Strategy 2) eTable 4. Regions Showing Significant Differences in fALFF Between Individuals With Lifetime Depression and the Single HC Group (Strategy 1) eTable 5. Regions Showing Significant Differences in LCOR Between Individuals With Lifetime Depression and the Single HC Group (Strategy 1) eTable 6. Regions Showing Significant Differences in GCOR Between Individuals With Lifetime Depression and the Single HC Group (Strategy 1) eTable 7. Regions Showing Significant Differences in GMV Between Individuals With Lifetime Depression and the Single HC Group (Strategy 1) eTable 8. Regions Showing Significant Differences in fALFF Between Individuals With Lifetime Depression and Matched HC Group (Strategy 2) eTable 9. Regions Showing Significant Differences in LCOR Between Individuals With Lifetime Depression and Matched HC (Strategy 2) eTable 10. Regions Showing Significant Differences in GCOR Between Individuals With Lifetime Depression and Matched HC (Strategy 2) eTable 11. Regions Showing Significant Differences in GMV Between Individuals With Lifetime Depression and Matched HC (Strategy 2) eReferences [file jamanetwopen-e2356787-s001.pdf]

Supplemental Online Content

Wang X, Hoffstaedter F, Kasper J, Eickhoff S, Patil KR, Dukart J. Lifetime exposure to depression and neuroimaging measures of brain structure and function. *JAMA Netw Open*. 2024;7(2):e2356787.
doi:10.1001/jamanetworkopen.2023.56787

eMethods.
eFigure 1. The Number of Participants in Criteria Constellations
eFigure 2. The Cerebral Parts of the Clusters Showing Significant Functional or Structural Differences Between Different Depression Stratums and HC
eFigure 3. The Cerebellum Parts of the Clusters Showing Significant Functional or Structural Differences Between Different Depression Stratums and HC
eTable 1. Demographic of Six Depression Groups Involving Analyses
eTable 2. Demographic of the Single Healthy Groups (Strategy 1)
eTable 3. Demographic of Six Matched Healthy Groups (Strategy 2)
eTable 4. Regions Showing Significant Differences in fALFF Between Individuals With Lifetime Depression and the Single HC Group (Strategy 1)
eTable 5. Regions Showing Significant Differences in LCOR Between Individuals With Lifetime Depression and the Single HC Group (Strategy 1)
eTable 6. Regions Showing Significant Differences in GCOR Between Individuals With Lifetime Depression and the Single HC Group (Strategy 1)
eTable 7. Regions Showing Significant Differences in GMV Between Individuals With Lifetime Depression and the Single HC Group (Strategy 1)
eTable 8. Regions Showing Significant Differences in fALFF Between Individuals With Lifetime Depression and Matched HC Group (Strategy 2)
eTable 9. Regions Showing Significant Differences in LCOR Between Individuals With Lifetime Depression and Matched HC (Strategy 2)
eTable 10. Regions Showing Significant Differences in GCOR Between Individuals With Lifetime Depression and Matched HC (Strategy 2)
eTable 11. Regions showing significant differences in GMV between individuals with lifetime depression and matched HC (Strategy 2)
eReferences

This supplemental material has been provided by the authors to give readers additional information about their work.

eMethods.

Six operational criteria for exposure to lifetime depression

(1) Help-seeking was responding “Yes” to the following questions: "Have you ever seen a general practitioner for nerves, anxiety, tension, or depression?" [Data-Field: 2090] or "Have you ever seen a psychiatrist for nerves, anxiety, tension, or depression?" [Data-Field: 2100].

(2) Self-reported Depression was having experienced depression at present or past [Data-Field ID: 20002] before the neuroimaging scan.

(3) The Antidepressant usage was taking antidepressant medication at baseline or follow-up assessment [Data-Field ID: 20003]. The antidepressant codes are listed in eMethod (Medications codes).

(4) Depression (Smith) is an approximate measure of lifetime depression by Smith.[1] Smith et.al defined three types of depression through relevant questions in the mental health questionnaire, including “probable single episode”, “probable mild recurrent”, and “probable severe recurrent”. Participants met one of three lifetime depression were defined as Depression (Smith).

(5) Hospital ICD-10 is the hospital recorder for patients’ primary and secondary diagnoses [Data-field ID: 41202 and 41204]. Patients with the diagnosis of the depressive episode (F32-F32.9) or recurrent depressive disorder (F33-F33.8) were included in this criteria.

(6) CIDI-SF is derived from the mental health questionnaire.[2] The CIDI-SF is a brief survey instrument design to identify mental disorders including MDD based on the Diagnostic and Statistical Manual of Mental Disorders (DSM) criteria.[3]

The exclusion criteria for lifetime depression

The exclusion criteria of individuals in the lifetime depression group were:

(1) Having self-reported psychosis. The self-reported psychosis was defined as having experienced schizophrenia or mania/bipolar disorder/manic depression at present or past [Data-Field ID: 20002].
(2) Having taken antipsychotic medications. The antipsychotic codes were listed in supplementary materials.
(3) The hospital records [Data-Field ID: 41202 or 41204] included Schizophrenia, schizotypal and delusional disorders (F20-F29), and other mood disorders (F30-F39, excluding depression code: F32-F33).
(4) Having been defined as psychosis in mental health questionnaire (MHQ) screen. The answer to the question “Have you been diagnosed with one or more of the following mental health problems by a professional, even if you don’t have it currently” [Data-field ID: 20544] is “Schizophrenia” or “Any other type of psychosis or psychotic illness”, or “Mania, hypomania, bipolar or manic-depression”.

© 2024 Wang X et al. *JAMA Network Open*

The exclusion criterion for healthy controls

The healthy individuals were enrolled with the following exclusive criterion:

(1) Did not meet the criteria for any indications of depression as described in the above depression phenotypes.
(2) Did not meet the criteria for any indications of psychosis which was described in the depression exclusive criterion, including self-reported psychosis, antipsychotic medications usage, MHQ psychosis.
(3) Did not endorse disorder in any mental illness and behavior disorder (Hospital ICD10 Chapter V, F00-F99) and diseases of the nervous system (Hospital ICD10 Chapter VI G00-G99）.

Medications codes

The antidepressants are coded as follows [UKB Data-Coding 4]:
1140879616, 1140921600, 1140879540, 1140867878, 1140916282, 1140909806, 1140867888, 1141152732, 1141180212, 1140879634, 1140867876, 1140882236, 1141190158, 1141200564, 1140867726, 1140879620, 1140867818, 1140879630, 1140879628, 1141151946, 1140867948, 1140867624, 1140867756, 1140867884, 1141151978, 1141152736, 1141201834, 1140867690, 1140867640, 1140867920, 1140867850, 1140879544, 1141200570, 1140867934, 1140867758, 1140867914, 1140867820, 1141151982, 1140882244, 1140879556, 1140867852, 1140867860, 1140917460, 1140867938, 1140867856, 1140867922, 1140910820, 1140882312, 1140867944, 1140867784, 1140867812, 1140867668.

The antipsychotics in UK biobank are coded as follows:
1140868170, 1140928916, 1141152848, 1140867444, 1140879658, 1140868120, 1141153490, 1140867304, 1141152860, 1140867168, 1141195974, 1140867244, 1140867152, 1140909800, 1140867420, 1140879746, 1141177762, 1140867456, 1140867952, 1140867150, 1141167976, 1140882100, 1140867342, 1140863416, 1141202024, 1140882098, 1140867184, 1140867092, 1140882320, 1140910358, 1140867208, 1140909802, 1140867134, 1140867306, 1140867210, 1140867398, 1140867078, 1140867218, 1141201792, 1141200458, 1140867136, 1140879750, 1140867180, 1140867546, 1140928260, 1140927956.

Imaging data acquisition and pre-processing

The present study focused on resting-sate functional MRI and T1-weight structural images in initial neuroimaging scans of these patients in the UK biobank. MRI data were acquired using a Siemens Skyra 3T scanner (Siemens Healthcare, Erlangen, Germany) using a standard 32-channel head coil, according to a freely available protocol (http://www.fmrib.ox.ac.uk/ukbiobank/protocol/V4_23092014.pdf). As part of the scanning protocol, high-resolution T1-weighted images and resting-state fMRI were obtained. High-resolution T1-weighted images were obtained using an MPRAGE sequence with the following parameters: repetition time (TR) =2000ms, echo time (TE) =2.01ms, 208 slices, flip angle=8°, field of view (FOV) =256mm, matrix=256×256, slice thickness=1.0mm, voxel size 1×1×1mm. The resting-state functional MRI were obtained with with a multi-band gradient echo EPI sequence: TR=735ms;

© 2024 Wang X et al. *JAMA Network Open*

TE=39ms; 64 slices; flip angle=52°; FOV=210mm; matrix=88×88; slice thickness=2.4mm, voxel size=2.4×2.4×2.4mm^3. Images that did not pass the existing UKB preprocessing and quality control pipeline[4] were excluded.

For T1-weight images, raw T1 images were registered to functional images, bias and noise corrected, global intensity normalized, and then segmented into gray matter (GM), white matter, and cerebrospinal fluid. Next, the images were spatially normalized to the standard Montreal Neurological Institute (MNI) templates using Geodesic Shooting.[5] A whole-brain gray matter mask with a probability of gray matter above 0.3 was applied prior to the analyses.

For functional images, we used the resting-state fMRI processed by the UK Biobank as "filtered_func_data_clean.nii" (https://biobank.ctsu.ox.ac.uk/crystal/crystal/docs/brain_mri.pdf). Functional images were processed by motion correction, grand-mean intensity normalization, high-pass temproal filtering, echo planar imaging unwarping, gradient distortion correction unwarping, and the removal of structural artefacts. Images were then co-registered to the corresponding high-resolution T1 anatomical images which were transformed into the MNI space. The resulting images were resampled to 3×3×3 mm^3 voxels, smoothed with a 4-mm-full-width, half-maximum Gaussian kernel. We discarded the first five functional time points to ensure signal equilibrium. Subsequently, temporal band-pass filtering (0.008-0.09 Hz) was performed (not for caculating the fractional amplitude of low-frequency fluctuations). Motion parameters (Friston 24 motor parameters)[6], average white matter and average cerebrospinal fluid signals were regressed out. We exclued the subjects with excessive head movement (frame-wise displacement > 3 mm and rotation > 2 °).

© 2024 Wang X et al. *JAMA Network Open*

eFigure 1. The Number of Participants in Criteria Constellations

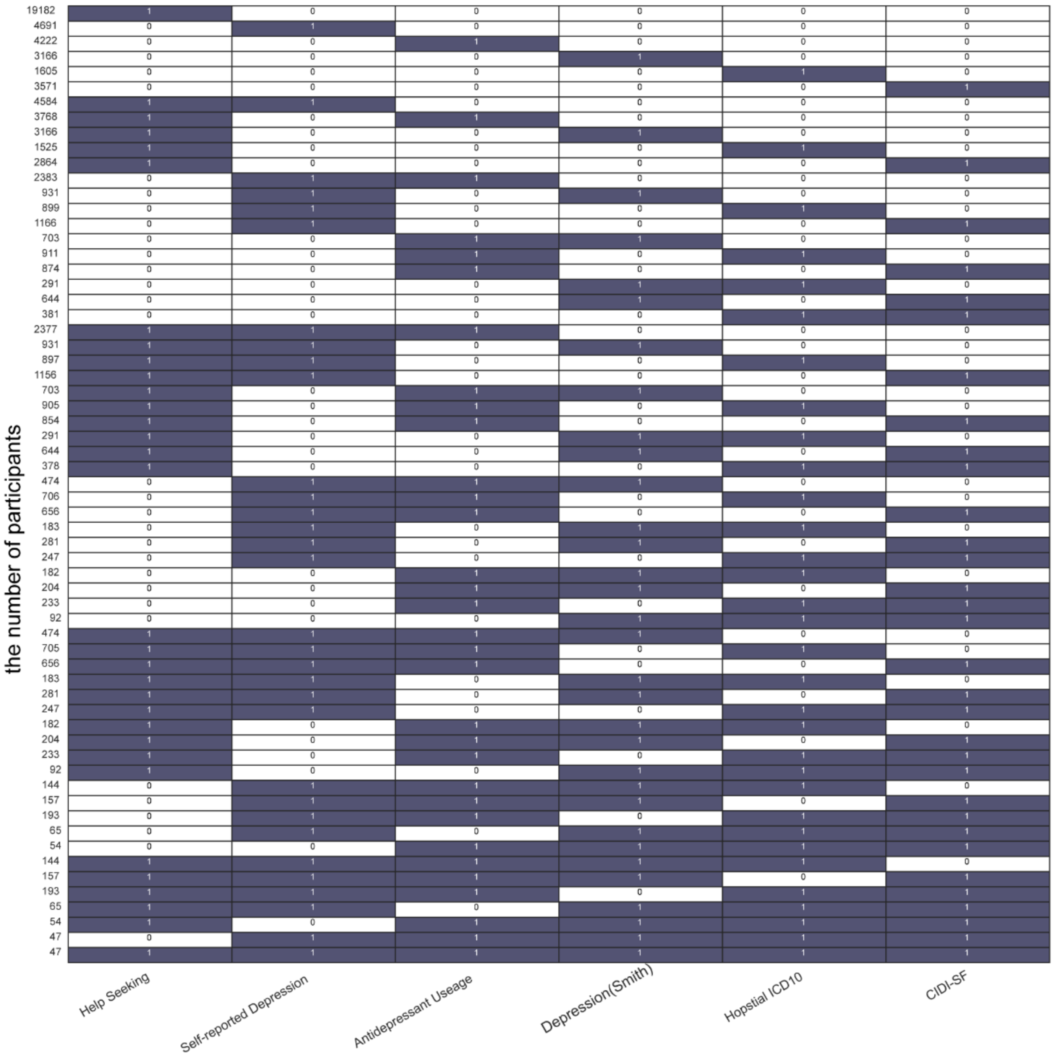

The first column indicates the number of participants in each cumulative constellation from meeting at least one criterion to meeting all six criteria. The tabular part indicates which criteria are involved. The dark element means that the current column contains corresponding criteria.

© 2024 Wang X et al. *JAMA Network Open*

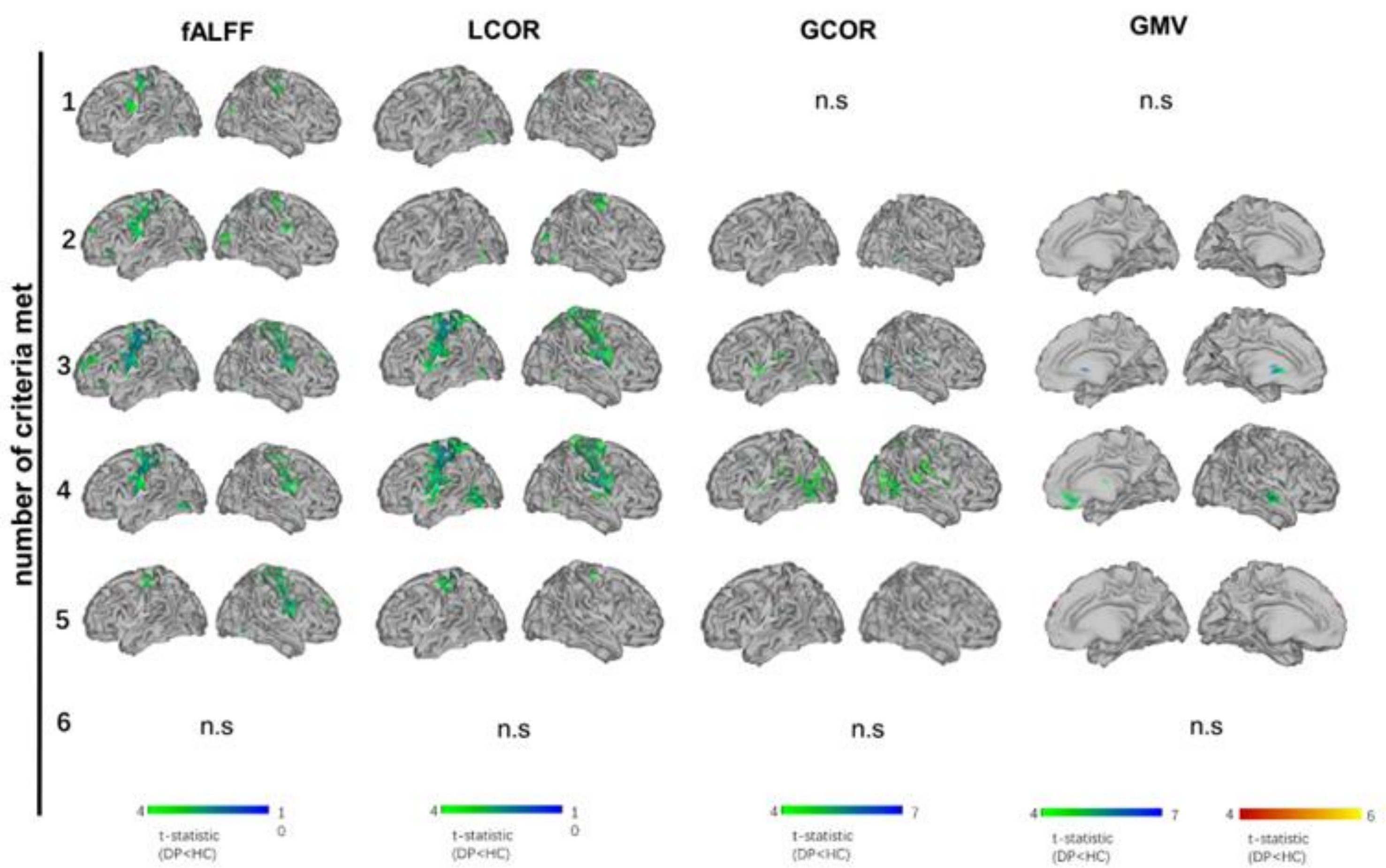

eFigure 2. The Cerebral Parts of the Clusters Showing Significant Functional or Structural Differences Between Different Depression Stratums and HC.

© 2024 Wang X et al. *JAMA Network Open*

The n.s indicates no significant clusters. Abbreviations: fALFF: fractional amplitude of low-frequency fluctuations. GCOR: global correlation, LCOR: local correlation, GMV: gray matter volumes.

© 2024 Wang X et al. *JAMA Network Open*

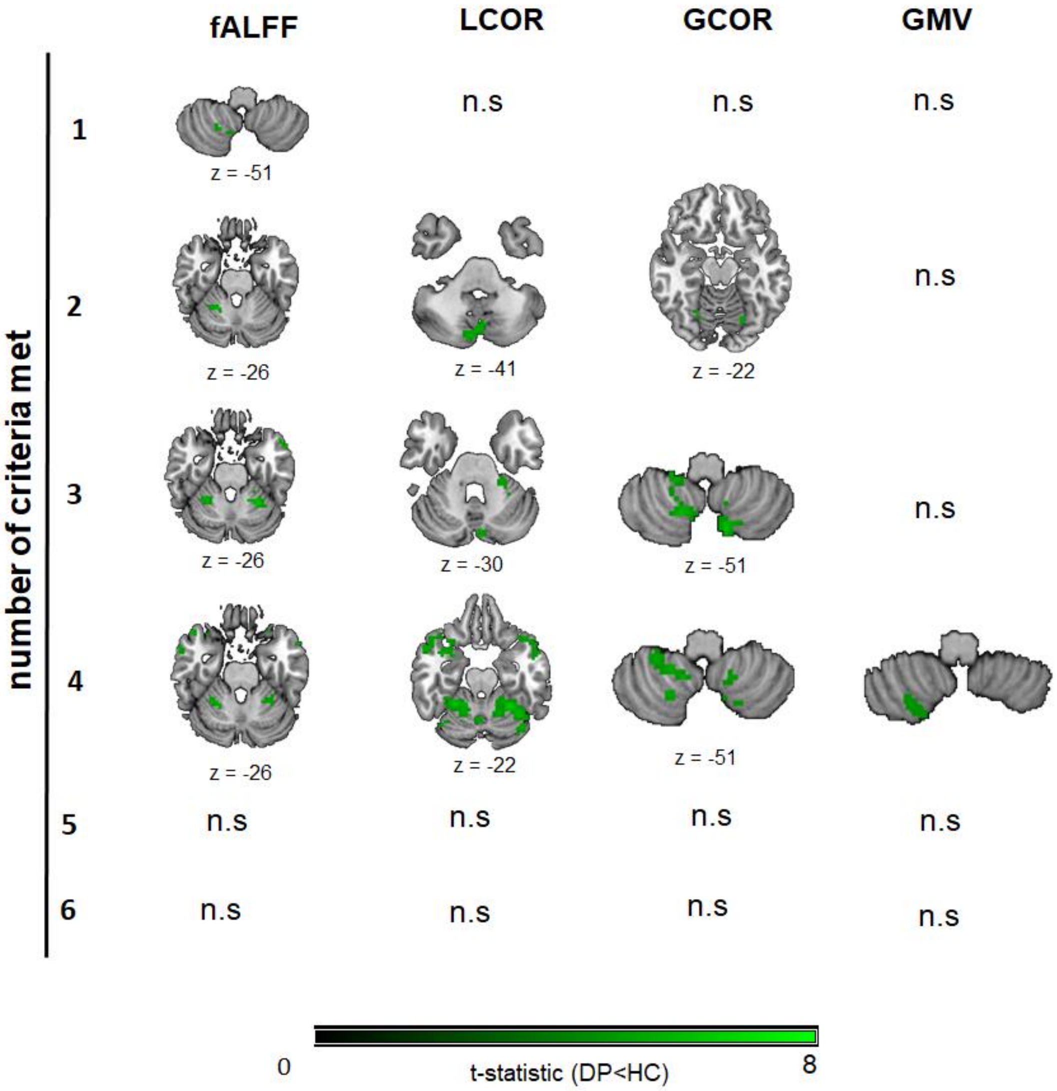

eFigure 3. The Cerebellum Parts of the Clusters Showing Significant Functional or Structural Differences Between Different Depression Stratums and HC

The n.s indicates the no significant difference in the cerebellum. Abbreviations: fALFF: fractional amplitude of low-frequency fluctuations. GCOR: global correlation, LCOR: local correlation, GMV: gray matter volumes.

© 2024 Wang X et al. *JAMA Network Open*

eTable 1. Demographic of Six Depression Groups Involving Analyses

Modality	N criteria met	Gender (female/male)	Age (years)	Education (years)
Function	1	3936/2572	64.01±7.4	16.59±3.84
	2	1971/1121	63.07±7.36	16.83±3.7
	3	1054/530	62.74±7.46	16.78±3.7
	4	530/238	61.94±7.43	16.73±3.8
	5	174/70	61.31±7.76	17.44±3.23
	6	19/10	59.82±7.16	17.48±2.71
Structure	1	5183/3419	63.99±7.38	16.58±3.85
	2	2628/1482	63.09±7.34	16.78±3.73
	3	1377/681	62.64±7.38	16.82±3.69
	4	706/335	62.03±7.41	16.85±3.71
	5	219/88	61.49±7.69	17.33±3.32
	6	27/12	59.09±7.06	17.77±2.45

© 2024 Wang X et al. *JAMA Network Open*

eTable 2. Demographic of the Single Healthy Groups (Strategy 1)

	Gender (female/male)	Age (years)	Education (years)
Initial HC group	11384/14078	65.05±7.8	16.69±3.77
Function	6606/7723	64.46±7.58	16.77±3.7
Structure	9263/11070	64.48±7.61	16.75±3.72

© 2024 Wang X et al. *JAMA Network Open*

eTable 3. Demographic of Six Matched Healthy Groups (Strategy 2)

Modalities	Healthy controls			
	N criteria met	Gender (female/male)	Age (mean±std)	Years of Education (mean±std)
Function	1	3945/2573	63.76±7.39	16.8±3.69
	2	1976/1120	63.05±7.31	16.87±3.68
	3	1053/534	62.66±7.44	16.86±3.61
	4	529/236	61.93±7.44	16.98±3.56
	5	172/71	61.39±7.4	17.59±2.97
	6	19/10	59.85±7.2	17.52±2.43
Structure	1	5184/3418	63.89±7.41	16.68±3.79
	2	2630/1480	63.13±7.32	16.91±3.63
	3	1375/683	62.63±7.36	16.9±3.59
	4	707/334	62.06±7.38	16.9±3.64
	5	220/87	61.6±7.62	17.36±3.23
	6	28/11	59.49±6.81	17.9±2.19

© 2024 Wang X et al. *JAMA Network Open*

eTable 4. Regions Showing Significant Differences in fALFF Between Individuals With Lifetime Depression and the Single HC Group (Strategy 1)

N criteria me	Contrast	Cluster size	Cluster p-value	Peak T statistic	Peak effect size	MNI coordinates	Anatomical region
1	DP<HC	43	p<.001	6.440	-0.109	[-51;27;-12]	left posterior orbital gyrus
		34	p<.001	6.407	-0.097	[-21;-84;45]	left superior temporal cortex
		31	p<.001	6.373	-0.097	[-63;-51;24]	left supraMarginal gyrus and middle temproal cortex
		49	p<.001	6.142	-0.103	[24;-48;-27]	right Lobule IV, V, VI of cerebellar hemisphere
2	DP<HC	55	p<.001	6.208	-0.134	[48;-69;-12]	right inferior temporal and occipital cortex
		156	p<.001	6.181	-0.139	[-33;48;33]	left middle and superior frontal cortex
		49	p<.001	6.027	-0.133	[21;-51;-27]	righ Lobule IV, V, VI of cerebellar hemisphere
		69	p<.001	5.969	-0.150	[-21;-57;-48]	left Lobule VIIB, VIII of cerebellar hemisphere
3	DP<HC	5545	p<.001	9.552	-0.250	[-42;-33;54]	bilateral medial and lateral pre- and postercentral gyrus
		271	p<.001	7.546	-0.220	[15;-60;-51]	bilateral loublue VIII and IX of cerebellar
		712	p<.001	7.494	-0.220	[-36;48;30]	left superiror medial frontal cortex
		318	p<.001	6.671	-0.205	[27;42;27]	right superior and medial frontal cortex
		156	p<.001	6.580	-0.222	[36;30;-12]	right inferior frontal gyrus
		63	p<.001	6.463	-0.198	[-21;-39;-24]	Left Lobule IV, V,VI of cerebellar hemisphere
		161	p<.001	6.362	-0.167	[-18;-102;15]	left middle and superiror occipital cortex
		90	p<.001	6.303	-0.191	[21;-51;-24]	right Lobule IV, V,VI of cerebellar hemisphere
		56	p<.001	6.168	-0.171	[48;-66;-12]	right inferior temporal gyrus
		92	p<.001	6.135	-0.191	[-57;-63;12]	left middle temporal gyrus
		47	p<.001	6.069	-0.174	[3;54;0]	right superior and medial frontal cortex

		29	p<.001	5.817	-0.149	[21;-96;24]	right occipital cortex
		25	p<.001	5.615	-0.162	[-21;-66;57]	left superior parietal cortex
4	DP<HC	4012	p<.001	9.061	-0.317	[-3;-9;51]	bilateral pre- and postcentral gyrus
		79	p<.001	7.045	-0.238	[-12;-99;24]	left superior and middle occipital cortex
		198	p<.001	6.419	-0.279	[-45;-66;-9]	left middle and inferior occipital cortex
		74	p<.001	6.411	-0.265	[24;-51;-21]	right Lobule IV, V,VI of cerebellar hemisphere
		60	p<.001	6.357	-0.239	[-9;66;24]	left superior frontal cortex
		57	p<.001	6.331	-0.276	[-6;-75;-45]	left Lobule VII, VIII of cerebellar hemisphere
		30	p<.001	6.327	-0.215	[12;-99;21]	right superior occipital cortex
		55	p<.001	6.216	-0.237	[39;57;15]	right superior and middle frontal cortex
		48	p<.001	6.162	-0.285	[-24;0;-12]	left amygdala and putamen and putamen
		36	p<.001	6.058	-0.250	[12;-69;-48]	right Lobule VIII of cerebellar hemisphere
		33	p<.001	6.006	-0.265	[24;3;-12]	right amygdala
		44	p<.001	5.821	-0.271	[36;21;-33]	right superior and middle temporal cortex
		38	p<.001	5.738	-0.245	[-18;-54;-18]	left Lobule IV, V of cerebellar hemisphere
		27	p<.001	5.568	-0.240	[-48;21;-15]	left posterior orbital gyrus and superior tempral pole cortex
		39	p<.001	5.511	-0.226	[45;-69;-9]	right fusiform
		28	p<.001	5.465	-0.243	[-57;9;-24]	left middle temporal cortex
5	DP<HC	312	p<.001	6.700	-0.410	[-42;-36;63]	left pre-and postcentral gyrus

© 2024 Wang X et al. *JAMA Network Open*

eTable 5. Regions Showing Significant Differences in LCOR Between Individuals With Lifetime Depression and the Single HC Group (Strategy 1)

N criteria met	Contrast	Cluster size	Cluster p-value	Peak T statistic	Peak effect size	MNI coordinates	Anatomical region
1	DP<HC	230	p<.001	6.686	-0.088	[0;-18;72]	left paracentral loubel and support motor area
		299	p<.001	6.329	-0.086	[39;-18;66]	right pre- and postcentral gyrus
		258	p<.001	6.216	-0.087	[-48;-27;60]	left pre-and postcentral gyrus
		34	p<.001	6.032	-0.084	[-9;-102;18]	left superior occipital cortex
		112	p<.001	5.487	-0.085	[36;-84;27]	right middle occipital cortex
		41	p<.001	5.281	-0.083	[-51;-72;-9]	left inferior temporal cortex
2	DP<HC	1155	p<.001	6.990	-0.129	[30;-15;63]	right pre- and postcentral gyrus
		82	p<.001	6.232	-0.130	[3;-75;-42]	left Crus II, Lobule VIII of cerebellar hemisphere
		87	p<.001	6.036	-0.124	[48;-66;-15]	right inferior occipital and temporal cortex
		122	p<.001	5.996	-0.131	[12;-57;-51]	right Lobule VIII, IX, X of cerebellar hemisphere
		356	p<.001	5.991	-0.123	[-33;-21;63]	left pre-and postcentral gyrus
		154	p<.001	5.830	-0.121	[6;-96;18]	left middle and superior occipital cortex
		175	p<.001	5.826	-0.110	[30;-87;33]	right superior and middle occipital cortex
		86	p<.001	5.629	-0.119	[-54;-75;3]	left inferior occipital and temporal cortex
		27	p<.001	5.605	-0.110	[-48;33;30]	left triangular part of inferior frontal cortex
		30	p<.001	5.309	-0.102	[57;-12;36]	right pre-and postcentral gyrus
3	DP<HC	6979	p<.001	9.066	-0.228	[-45;-33;63]	bilateral pre- and postcentral gyrus
		594	p<.001	6.922	-0.203	[3;-72;-42]	bilateral Lobule VIII and IX of cerebellar heimsphere
		124	p<.001	6.619	-0.174	[-21;-48;-15]	left fusiform gyrus
		105	p<.001	5.984	-0.159	[48;-63;-12]	right inferior temporal and occipital cortex

© 2024 Wang X et al. *JAMA Network Open*

		60	p<.001	5.507	-0.155	[63;-57;9]	right middle temporal cortex
		105	p<.001	5.479	-0.160	[-33;30;0]	left triangular part of inferior frontal gyrus
		87	p<.001	5.463	-0.156	[-48;-66;-6]	left inferior temporal and occipital cortex
		41	p<.001	5.230	-0.144	[-54;-60;12]	left middle temporal gyrus
		52	p<.001	5.163	-0.161	[15;-36;-3]	right fusiform gyrus and lingual cortex
4	DP<HC	7633	p<.001	9.593	-0.313	[-48;-27;60]	bilateral pre-and postcentral gyrus
		1212	p<.001	7.797	-0.300	[-48;-69;-6]	left middle and inferior temproal cortex and bilateral fusiform gyrus
		423	p<.001	6.627	-0.264	[-3;-75;-42]	bilateral Lobule VIIB, and VIII of cerebellar hemisphere
		37	p<.001	5.891	-0.217	[-42;33;39]	left middle frontal cortex
		61	p<.001	5.707	-0.205	[-18;-99;21]	left superior occipital cortex
		29	p<.001	5.656	-0.216	[-24;0;-9]	left putamen and amygdala
		36	p<.001	5.618	-0.211	[42;27;36]	right middle frontal cortex
		38	p<.001	5.591	-0.205	[-6;39;24]	left superior medial frontal cortex and anterior cingulate cortex
		47	p<.001	5.444	-0.208	[39;-69;-48]	right Crus II, Lobule VIIB, and VIII of cerebellar hemisphere
5	DP<HC	464	p<.001	6.892	-0.394	[-45;-27;63]	left pre-and postcentral gyrus
		48	p<.001	6.043	-0.392	[-48;-21;21]	left ralandic operculum and supramarginal cortex
		193	p<.001	5.770	-0.384	[-3;-18;51]	bilateral support motor area
		46	p<.001	5.691	-0.360	[-63;-6;12]	left postcentral gyrus and superior temporal cortex
		255	p<.001	5.664	-0.361	[39;-30;48]	right pre- and postcentral gyrus
		106	p<.001	5.655	-0.363	[-12;-33;78]	bilateral paracentral lobule; bilateral pre- and postcentral cortex
		59	p<.001	5.357	-0.361	[66;-12;27]	Right postcentral gyrus

© 2024 Wang X et al. *JAMA Network Open*

eTable 6. Regions Showing Significant Differences in GCOR Between Individuals With Lifetime Depression and the Single HC Group (Strategy 1)

N criteria met	Contrast	Cluster size	Cluster p-value	Peak T statistic	Peak effect size	MNI coordinates	Anatomical region
2	DP<HC	28	p<.001	6.051	-0.113	[-54;-75;3]	left middle and inferior temporal cortex
		41	p<.001	5.850	-0.114	[57;-63;-3]	right middle and inferior temporal cortex
		33	p<.001	5.771	-0.111	[18;-54;-9]	right lingual and fusiform gyrus
		26	p<.001	5.457	-0.109	[-39;21;30]	left inferior frontal cortex
3	DP<HC	826	p<.001	7.483	-0.170	[-39;0;12]	left insula and superior temporal cortex
		2852	p<.001	7.071	-0.175	[-21;-45;-15]	left middle cingulate cortex, precuneus and lingual cortex
		669	p<.001	6.245	-0.162	[39;-3;15]	right insular and rolandic operculum
		192	p<.001	6.225	-0.182	[9;-69;-45]	bilateral Lobule VIII of cerebellar hemisphere
		43	p<.001	5.675	-0.150	[48;3;39]	right precentral cortex
		90	p<.001	5.408	-0.149	[-60;-18;42]	left inferior parietal superior cortex
		36	p<.001	5.283	-0.160	[39;6;-15]	right superior temporal cortex
		31	p<.001	5.225	-0.140	[-48;3;39]	left precentral cortex
4	DP<HC	7471	p<.001	7.684	-0.250	[9;-60;69]	bilateral precuneus, lingual cortex, fusiform, middle and superior temporal cortex
		148	p<.001	6.912	-0.231	[-36;3;33]	left precentral cortex
		191	p<.001	6.772	-0.249	[15;-66;-45]	bilateral Lobule VIII and left Lobule VIIB of cerebellar hemisphere

© 2024 Wang X et al. *JAMA Network Open*

		32	p<.001	6.419	-0.236	[-24;0;-12]	left amygdala and putamen
		52	p<.001	6.238	-0.241	[27;-42;-48]	right Lobule VIII of cerebellar hemisphere
		165	p<.001	5.824	-0.224	[39;9;33]	right precentral cortex and inferior frontal cortex
		28	p<.001	5.355	-0.199	[-33;-42;42]	left inferior parietal cortex
		25	p<.001	5.121	-0.198	[-36;-3;54]	left precentral cortex
5	DP<HC	59	p<.001	5.825	-0.361	[-48;-33;24]	left supramarginal gyrus and superior temproal cortex

© 2024 Wang X et al. *JAMA Network Open*

eTable 7. Regions Showing Significant Differences in GMV Between Individuals With Lifetime Depression and the Single HC Group (Strategy 1)

N criteria met	Contrast	Cluster size	Cluster p-value	Peak T statistic	Peak effect size	MNI coordinates	Anatomical region
2	DP>HC	56	p<.001	5.286	0.086	[12;-30;75]	right pre- and post-central gyrus
3	DP<HC	396	p<.001	6.855	-0.142	[3;4.5;3]	bilateral Olfactory cortex
	DP<HC	60	p<.001	5.038	-0.110	[18;-13.5;-10.5]	right hippocampus
4	DP<HC	1608	p<.001	6.285	-0.183	[0;33;-15]	bilateral medial frontal cortex
		218	p<.001	6.058	-0.185	[28.5;30;36]	right superior and medial frontal cortex
		1051	p<.001	5.761	-0.179	[58.5;-10.5;-9]	right superior and middle temporal cortex
		434	p<.001	5.568	-0.182	[-3;-16.5;49.5]	bilateral middle cingulate cortex
		147	p<.001	5.317	-0.162	[18;-16.5;-12]	right hippocampus
		70	p<.001	5.192	-0.160	[21;-45;-4.]	right lingual cortex and parahippocampus
		161	p<.001	5.129	-0.162	[-49.5;3;-3]	left superior temporal cortex
		391	p<.001	5.074	-0.176	[-49.5;3;-28.5]	left inferior and middle temporal cortex
		208	p<.001	5.036	-0.157	[0;-9;9]	bilateral media dorsal medial magnocellular
		113	p<.001	4.981	-0.163	[-45;-28.5;48]	left postcentral gyrus
		171	p<.001	4.921	-0.163	[1.5;37.5;25.5]	left anterior cingulate cortex
		84	p<.001	4.920	-0.158	[-58.5;-33;16.5]	left superior temporal
		112	p<.001	4.885	-0.160	[52.5; 3;0]	right insula and righ rolandic operculum
		215	p<.001	4.870	-0.153	[25.5;-63;-58.5]	right Lobule VIII of cerebellar hemisphere
		177	p<.001	4.856	-0.157	[43.5;-15;9]	right insula
		51	p<.001	4.714	-0.150	[30;12;-25.5]	right temporal pole: superior temporal gyrus
5	DP>HC	62	p<.001	4.670	0.259	[12;64.5;33]	right superior medial frontal cortex

© 2024 Wang X et al. *JAMA Network Open*

eTable 8. Regions Showing Significant Differences in fALFF Between Individuals With Lifetime Depression and Matched HC Group (Strategy 2)

N criteria met	Contrast	Cluster size	Cluster p-value	Peak T statistic	MNI coordinates	Anatomical region
2	DP<HC	537	p<.001	6.61	[27;-78;-6]	right Fusiform gyrus, lingual gyrus, occipital pole
		196	p<.001	5.91	[-39;-81;-9]	left middle occipital cortex
		75	p<.001	5.72	[-6;-21;66]	left thalamus
		46	p<.001	5.65	[-30;-9;60]	left precentral gyrus
3	DP<HC	2759	p<.001	8.05	[0;-30;63]	bilateral medial and lateral primary sensorimotor regions
		90	p<.001	6.46	[18;-60;-51]	right Lobule VIII of cerebellar hemisphere
		121	p<.001	6.17	[-39;-24;21]	left parietal operculum cortex
		70	p<.001	6.04	[0;60;27]	superior medial frontal
		115	p<.001	5.96	[-27;60;21]	left superior frontal cortex
		32	p<.001	5.93	[-30;-51;-48]	left Lobule VIII of cerebellar hemisphere
		28	p<.001	5.79	[0;54;-9]	left medial frontal cortex
		34	p<.001	5.72	[-9;-72;-48]	left Lobule VIII of cerebellar hemisphere
		30	p<.001	5.57	[42;-69;-12]	right lateral occipital cortex
		30	p<.001	5.55	[-27;-87; 0]	left middle occipital cortex
4	DP<HC	137	p<.001	7.63	[0;-9;51]	bilateral support motor regions
		683	p<.001	7.19	[-54;-12;33]	left postcentral gyrus
		280	p<.001	6.74	[60;-9;33]	right postcentral gyrus

© 2024 Wang X et al. *JAMA Network Open*

68	p<.001	6.13	[-3;-36;60]	left postcentral gyrus
32	p<.001	6.06	[12;54;39]	right superior medial frontal cortex
119	p<.001	5.92	[3;57;30]	right superior medial frontal cortex
46	p<.001	5.48	[-45;-69;-9]	left inferior occipital cortex

© 2024 Wang X et al. *JAMA Network Open*

eTable 9. Regions Showing Significant Differences in LCOR Between Individuals With Lifetime Depression and Matched HC (Strategy 2)

N criteria met	Contrast	Cluster size	Cluster p-value	Peak T statistic	MNI coordinates	Anatomical region
2	DP<HC	79	p<.001	6.22	[27;-48;-51]	right inferior cerebellum
		38	p<.001	5.77	[15;-45;-51]	right Lobule IX of cerebellar hemisphere
		259	p<.001	5.65	[33;-84;-12]	right inferior occipital
		31	p<.001	5.24	[0;-93;12]	calcarine
		31	p<.001	5.16	[-42;-75;-9]	left inferior occipital
3	DP<HC	2734	p<.001	6.94	[-3;-30;60]	bilateral medial and superior sensorimotor regions
		190	p<.001	6.61	[-12;-72;-51]	left Lobule VIII of cerebellar hemisphere
		168	p<.001	6.12	[15;-60;-51]	right Lobule VIII of cerebellar hemisphere
		45	p<.001	6.12	[-42;-6;3]	left insula
		50	p<.001	5.90	[-12;-57;-48]	left Lobule IX of cerebellar hemisphere
		27	p<.001	5.76	[-42;-51;-21]	left fusiform
		99	p<.001	5.54	[-15;-48;-3]	left lingual and fusiform, parahippocampus
		27	p<.001	5.35	[-39;-75;-12]	left fusiform
4	DP<HC	623	p<.001	6.77	[-42;-24;63]	bilateral medial and lateral sensorimotor regions
		172	p<.001	6.09	[63;-15;30]	postcentral gyrus
		66	p<.001	5.58	[0;-6;48]	left middle cingulate cortex
		64	p<.001	5.57	[-15;-45;75]	left precuneus
		55	p<.001	5.56	[51;-24;60]	right precentral gyrus

© 2024 Wang X et al. *JAMA Network Open*

		38	p<.001	5.53	[63;-9;-27]	right anterior middle temporal gyrus
		70	p<.001	5.52	[-51;-66;-6]	left inferior temporal cortex
5	DP<HC	92	p<.001	5.44	[-45;-21;63]	left postcentral gyrus

© 2024 Wang X et al. *JAMA Network Open*

eTable 10. Regions Showing Significant Differences in GCOR Between Individuals With Lifetime Depression and Matched HC (Strategy 2)

N criteria met	Contrast	Cluster size	Cluster p-value	Peak T statistic	MNI coordinates	Anatomical region
3	DP<HC	42	p<.001	5.43	[-24;-42;-3]	left lingual cortex and parahippocampus

eTable 11. Regions Showing Significant Differences in GMV Between Individuals With Lifetime Depression and Matched HC (Strategy 2)

N criteria met	Contrast	Cluster size	Cluster *p*-value	Peak T statistic	MNI coordinates	Anatomical region
2	DP>HC	56	0.002	5.14	[14;-33;75]	Right postcentral gyrus
3	DP<HC	152	0.004	5.64	[3;4;3]	Subcallosal cortex
4	DP<HC	218	0.003	5.06	[-44;-15;44]	left precentral gyrus
		64	0.015	4.87	[0;-17;50]	left precentral gyrus
		62	0.015	4.69	[0;32;-17]	Medial frontal cortex
5	DP<HC	129	0.094	5.52	[41;-24;48]	right postcentral cortex

© 2024 Wang X et al. *JAMA Network Open*

eReferences

1. Smith DJ, Nicholl BI, Cullen B, et al. Prevalence and characteristics of probable major depression and bipolar disorder within UK biobank: cross-sectional study of 172,751 participants. *PLoS One*. 2013;8(11):e75362. doi:10.1371/journal.pone.0075362
2. Davis KAS, Coleman JRI, Adams M, et al. Mental health in UK Biobank - development, implementation and results from an online questionnaire completed by 157 366 participants: a reanalysis. *BJPsych Open*. Feb 6 2020;6(2):e18. doi:10.1192/bjo.2019.100
3. American Psychiatric Association D, Association AP. *Diagnostic and statistical manual of mental disorders: DSM-5*. vol 5. American psychiatric association Washington, DC; 2013.
4. Alfaro-Almagro F, Jenkinson M, Bangerter NK, et al. Image processing and Quality Control for the first 10,000 brain imaging datasets from UK Biobank. *Neuroimage*. Feb 1 2018;166:400-424. doi:10.1016/j.neuroimage.2017.10.034
5. Ashburner J, Friston KJ. Diffeomorphic registration using geodesic shooting and Gauss-Newton optimisation. *Neuroimage*. Apr 1 2011;55(3):954-67. doi:10.1016/j.neuroimage.2010.12.049
6. Friston KJ, Williams S, Howard R, Frackowiak RS, Turner R. Movement-related effects in fMRI time-series. *Magn Reson Med*. Mar 1996;35(3):346-55. doi:10.1002/mrm.1910350312

© 2024 Wang X et al. *JAMA Network Open*
