# Supplementary material for: Lifetime Exposure to Depression and Neuroimaging Measures of Brain Structure and Function
Source: JAMA Netw Open. 2024 Feb 19;7(2):e2356787. doi: 10.1001/jamanetworkopen.2023.56787 (PMC10877455; doi:10.1001/jamanetworkopen.2023.56787)
Supplement: Supplement 2. — Data Sharing Statement [file jamanetwopen-e2356787-s002.pdf]

Data Sharing Statement

Wang. Lifetime Exposure to Depression and Neuroimaging Measures of Brain Structure and Function. *JAMA Netw Open*. Published February 19, 2024.
doi:10.1001/jamanetworkopen.2023.56787

Data
Data available: No

Additional Information
Explanation for why data not available: The images and phenotypic data that support the current findings are available from UK Biobank. Data would be available from the authors upon reasonable application and with permission of UK Biobank (http://www.ukbiobank.ac.uk/).
